# Differences in Delay, but not Probability Discounting, in Current Smokers, E-cigarette Users, and Never Smokers

**DOI:** 10.1007/s40732-017-0244-1

**Published:** 2017-04-24

**Authors:** Wojciech Białaszek, Przemysław Marcowski, David J. Cox

**Affiliations:** 1 SWPS University of Social Sciences and Humanities, Chodakowska 19/31, 03-815 Warsaw, Poland; 2 University of Florida, + 945 Center Drive, Gainesville, FL 32611-2250 USA

**Keywords:** E-cigarette, Smoking, Delay discounting, Probability discounting, Risk in delay

## Abstract

Steeper delay discounting in substance abuse populations, compared to non-abusing populations, has been well-established in prior studies. Despite the growing interest in e-cigarettes as a novel and relatively understudied form of nicotine consumption, relatively little is known as to how e-cigarette users discount rewards compared to traditional cigarette smokers and never smokers. In the present study, we measured delay and probability discounting rates, as well as perceived risk inherent to a delayed reward, in current traditional cigarette smokers, e-cigarette users, and never smokers. We found that traditional cigarette smokers and e-cigarette users discounted delayed rewards at a similar rate—and both were steeper than never smokers. However, no differences were observed in probability discounting or in perceived risk inherent in reward delay.

Nicotine addiction is a major contemporary global health concern. Various forms of consumption and widespread availability make it one of the most common addictions worldwide and a leading behavioral cause of premature death (Sussman, Lisha, & Griffiths, 2011). In addition, the World Health Organization (WHO) estimates the global prevalence of smoking tobacco use is 36.1% in males and 6.1% in females, reaching prevalence estimates as high as 48.5% in certain regions of the globe (WHO, 2016).

Traditionally, and most commonly, nicotine ingestion occurs through smoking tobacco. However, other forms of nicotine consumption are also widely used. An increasingly popular and more recent method of nicotine consumption is e-smoking (Ayers, Ribisl, & Brownstein, 2011; Goniewicz, Gawron, Nadolska, Balwicki, & Sobczak, 2014; Kośmider, Knysak, Goniewicz, & Sobczak, 2012). Electronic cigarettes (e-cigarettes) are electronically operated devices that vaporize liquid that contains nicotine. The vapor is then inhaled in a manner similar to smoking traditional cigarettes. Given the relative novelty of e-cigarettes, the potential short- and long-term risks of nicotine consumption through vaporized liquid is not yet fully known (Goniewicz et al., 2016). Relatedly, little is known regarding behavioral patterns of e-cigarette users compared to traditional cigarette smokers. In the past few years, the popularity of e-smoking has rapidly increased not only in Poland (Goniewicz, et al., 2014), but also worldwide (Dockrell, Morison, Bauld, & McNeill, 2013; McMillen, Gottlieb, Shaefer, Winickoff, & Klein, 2015), and it is important to explore this new group of nicotine consumers.

The discounting paradigm serves as a powerful platform for researchers interested in understanding substance use and abuse. Difference (or their absence) in rates of discounting between substance abusers and non-abusers provides a theoretical framework for understanding why substance abuse occurs and is maintained over time, which may lead to addiction (Mitchell, 2004). The term discounting refers to the observation that the present value of a consequence decreases as a function of the delay to its receipt (e.g., Mazur, 1987) or as a function of its decreasing probability of occurrence (e.g., Rachlin, Raineri, & Cross, 1991).

The relevance of the discounting paradigm to addiction has led previous researchers to study the relation between the presence/absence of addiction and rates of delay discounting (Amlung, Vedelago, Acker, Balodis, & MacKillop, 2016; Barlow, McKee, Reeves, Galea, & Stuckler, 2016). Recent meta-analyses of delay discounting and addictive behavior have found that the relation is robust. Addiction is accompanied by steeper delay discounting (Amlung et al., 2016; MacKillop et al., 2011). In addition, and perhaps more importantly, delay discounting has been shown to provide a conceptual account of the emergence and maintenance of addiction itself (for a review, see Mitchell, 2004).

Several researchers have specifically compared delay and probability discounting relative to smoking and non-smoking status. For example, Mitchell (1999) found that smokers discounted delayed outcomes more steeply than non-smokers. However, smokers and non-smokers did not differ in rates of probability discounting. Ohmura, Takahashi, and Kitamura (2005) also reported differences in delay discounting but no differences in probability discounting between never and light to moderate smokers. In contrast, Reynolds, Richards, Horn, and Karraker (2004) showed that both measures of delay and probability discounting were predictors of smoking status. However, they note that the effect in delay discounting was significantly greater than the effect in probability discounting.

To the best of our knowledge, only two studies have measured delay discounting in e-cigarette users (Weidberg, González-Roz, & Secades-Villa, 2016; Chivers, Hand, Priest, & Higgins, 2016). Weidberg and colleagues (2016) compared delay discounting in e-cigarette users, traditional cigarette smokers, former smokers, and non-smoking controls. They found that e-cigarette users discounted delayed rewards more steeply than former smokers, but did not differ from current smokers and controls. In addition, cigarette smokers discounted delayed rewards more steeply than former smokers and controls but did not differ from e-cigarette users. Chivers and colleagues (2016) examined e-cigarette use in 800 women of reproductive age. While their study primarily sought to examine associations between risk factors and e-cigarette use, they asked participants to complete several measures of impulsivity including a delay discounting task. The authors observed significantly different rates of discounting between current cigarette smokers and never smokers but did not observe a difference between e-cigarette users and never smokers nor between smokers and e-cigarette users. Together, these data indicate that e-cigarette users show delay discounting rates somewhere between current cigarette smokers and current non-smokers.

Other measures of risk seeking and impulsivity may also differentiate current cigarette smokers from e-cigarette users. Given previous researchers have examined rates of probability discounting as a function of smoking status, comparing rates of probability discounting between e-cigarette users, current smokers, and never smokers seems warranted. Previous researchers found no difference between cigarette smokers and non-smokers and a similar pattern is likely to be observed with e-smokers. Nevertheless, until tested directly, the relationship between probability discounting and e-cigarette users is unknown.

Another measure relevant to smoking populations is perceived riskiness of delayed payoffs. Studies examining perceived riskiness of delayed payoffs attempt to determine how participants rate the uncertainty associated with delayed rewards in a delay-discounting assessment (e.g., Patak & Reynolds, 2007). One hypothesis is that delay discounting may be a byproduct of future uncertainty. That is, if one perceives the future as uncertain, they discount delayed outcomes more steeply than a person certain of the future occurrence of the same event. To our knowledge, only one study has investigated the relation of perceived riskiness of delayed payoffs to smoking status. Reynolds, Patak, and Shroff (2007) demonstrated that adolescent smokers rated delayed rewards as less certain than non-smokers, and this result was also accompanied by steeper delay discounting. These results, however, were small in size and the participants consisted of adolescents. Thus, replication of the results is needed with adult smokers. Finally, these authors did not measure probability discounting and its relation to perceived riskiness in smoking populations.

The present study aims to investigate delay and probability discounting as well as perceived riskiness of delayed payoffs in three groups of participants: never smokers, traditional cigarette smokers, and e-cigarettes users. We predict cigarette smokers and e-smokers will discount delayed rewards more steeply than never smokers. Second, given previous research on probability discounting between smokers and non-smokers, we predict there will be no difference in probability discounting between the three groups. Reynolds, Patak, and Shroff (2007) demonstrated that adolescent smokers perceive the future as more uncertain than non-smokers. As a result, we predict that the subjective probability of obtaining future rewards will be lower in e-smokers and smokers compared to never smokers. That is, both smoking groups should display higher uncertainty in the estimation of obtaining future rewards. This would be observed as higher subjective probability questionnaire (SPQ) scores in smokers and e-smokers, compared to never smokers. Last, previous research suggests we should observe a positive correlation between delay and probability discounting, as well as between delay discounting and SPQ scores (Białaszek, Gaik, McGoun, & Zielonka 2015; Sozou, 1998; Takahashi, Ikeda, & Hasegawa, 2007).

## Method

### Participants

A total of 126 undergraduate students (47 male and 79 female; 23.3 ± 5.3, mean age ± SD) were recruited for the study in accordance with SWPS University (Faculty of Psychology) ethics committee regulations. All participants were recruited by direct personal recruitment among the students of the university, and by an internet ad posted on a website where students could sign up for research participation. Informed consent was obtained from all individual participants included in the study. Basic sociodemographic information was collected, including data on smoking or use of e-cigarettes. Each participant completed a Fagerström Test for Nicotine Dependence (FTND; Heatherton, Kozlowski, Frecker, & Fagerström, 1991) that was modified for participants in the e-cigarette user group (Etter & Eissenberg, 2015). For e-cigarette users, we substituted the word “cigarette” with “e-cigarette” and defined single use of an e-cigarette as taking at least 15 puffs or an e-smoking event lasting at least 10 minutes (Etter & Eissenberg, 2015; see also Foulds et al. 2015).

Out of all recruited participants, 90 met inclusion criteria and were included in the analysis (36 male and 54 female; 23.7 ± 5.9, mean age ± SD). There were six inclusion criteria. The first two were: (1) producing systematic discounting, i.e., each subsequent value discounted by an increase in delay or odds against receiving a reward did not exceed a 20% increment over the previous one, starting from the second indifference point; and (2) the indifference value for the final delay or odds against receiving a reward was not greater than the first discounted value. These two criteria were based on Johnson and Bickel’s (2008) algorithm (see also White, Redner, Skelly, & Higgins, 2015). Non-discounting inclusion criteria were: (3) all never smokers declared to have never taken up smoking or to have used e-cigarettes regularly prior to the study and, therefore, they also declared no attempts of quitting smoking (because they have never smoked); (4) all cigarette smokers and all e-cigarette users, respectively, declared to be smoking only traditional cigarettes or only using e-cigarettes at the time of the study; (5) never smokers, traditional cigarette smokers, or e-cigarette users declared not to be in the process of quitting smoking; (6) all participants declared no other addictions (one participant, however, claimed to be addicted to chocolate but was included in the sample).

As illustrated in Table 1, groups of traditional cigarette smokers, e-cigarette users, and never smokers did not differ with respect to sex composition (*p* = 0.647) and age (*p* = 0.790). We did observe significantly higher FTND scores in e-cigarette users compared to cigarette smokers [*F*(1, 56) = 8.023; *p* = 0.006; *η*_p_^*2*^ = 0.13]. In addition, cigarette and e-cigarette users had smoked for a median of 60 and 35 months prior to the start of the study, respectively. The number of cigarettes smoked or e-cigarette uses per day were also collected from participants. These were defined as smoking a single cigarette in the traditional cigarette smokers group, and in the e-cigarette users as taking at least 15 puffs or using an e-cigarette for at least 10 minutes. In the traditional cigarette smokers group, half of the participants smoked more than 5 cigarettes per day and half of the e-smokers used an e-cigarette over 10 times a day. Most commonly, traditional cigarette smokers smoked light-type cigarettes (72% of cases), followed by full-flavor cigarettes (24%), and ultra-light (4%). In the e-smokers, participants most commonly used a liquid containing 12 mg/ml of nicotine (43% of cases), followed by 18 mg/ml liquid (25%), 6 mg/ml (14%), and 9 mg/ml and 24 mg/ml (4% in both cases). The remaining participants used a liquid with other nicotine content (10%).

**Table 1:** Characteristics of traditional cigarette smokers, e-cigarette users, and never smokers

Variable	Cigarrete smokers (n = 29)	e-Cigarette users (n = 30)	Never smokers (n = 31)	Statistic value	*p*
Sex					
n Male	11	14	11	$X$ ^2^(2) = 0.87	0.647
n Female	18	16	20		
Age					
Mean ± SD	23.38 ± 5.09	24.27 ± 6.25	23.32 ± 6.46	F(2, 87) = 0.24	0.790
FTND Score					
Mean ± SD	2.00 ± 2.18	3.97 ± 3.01	-	F(1, 52.80) = 8.20	0.006

## Procedure

After participants signed an informed consent document, provided all demographic information, and provided information related to smoking status, they completed a paper-and-pencil questionnaire with two parts. First, participants completed delay and probability discounting tasks based on a fixed choice procedure with titrating values (Rachlin, Raineri, & Cross, 1991). The two tasks were presented in counterbalanced order across participants. We included five delay conditions (1, 12, 36, 60, and 120 months) and five probability conditions (95%, 80%, 55%, 20%, and 5%) of obtaining a hypothetical monetary reward of PLN 4500 (PLN 1 was equivalent to approximately USD 0.20 at the time of the study). Discounting for each delay or probability of obtaining a reward was measured on a separate pages of the questionnaire. For each discounting measure, participants were presented with a series of delayed or probabilistic hypothetical monetary payoffs (right column) and their immediate or certain alternatives (left column). The value of delayed or probabilistic payoffs was held constant, while their immediate or certain alternatives were presented in descending order from PLN 4500 to PLN 0 over 32 decrements for each delay or probability of obtaining a reward. In each corresponding row of both columns, the participants had to indicate their preference between delayed or probabilistic (right column) and immediate or certain alternatives (left column). This procedure identifies the value where participant preference shifts from immediate or certain payoffs to the delayed or probabilistic alternatives. This value was then used to infer an indifference point (i.e., the immediate or certain value that is equivalent to the delayed or probabilistic value)—which is also the subjective value of a given delayed or probabilistic reward.

Second, participants completed an adapted SPQ task (Takahashi, Ikeda, & Hasegawa, 2007). The task was used to assess the degree of perceived risk inherent to delay. Participants were asked to estimate the probability of obtaining a reward of PLN 4500 that is delayed by 1, 12, 36, 60, and 120 months (which were equal to the delays from the discounting task). No other information as to payoff source was provided. Estimates were given via a fill-in-the-blanks method, i.e., by indicating the level of subjective certainty percentage (0% to 100%) of obtaining a reward with a given delay time. At the end of the study, relevant groups completed the FTND test.

## Results

Area under the curve (AUC) was used as the dependent measure for discounting tasks (Myerson, Green, & Warusawitharana, 2001). Higher AUC values correspond with shallower discount rates and lower AUC values correspond to steeper discount rates. Separate one-way between-subject analyses of variance (ANOVAs) were conducted for rate of delay discounting, rate of probability discounting, and the subjective probability of obtaining delayed rewards (measured by SPQ) across the groups of never smokers, current e-cigarette users, and traditional cigarette smokers.

**Fig. 1:**
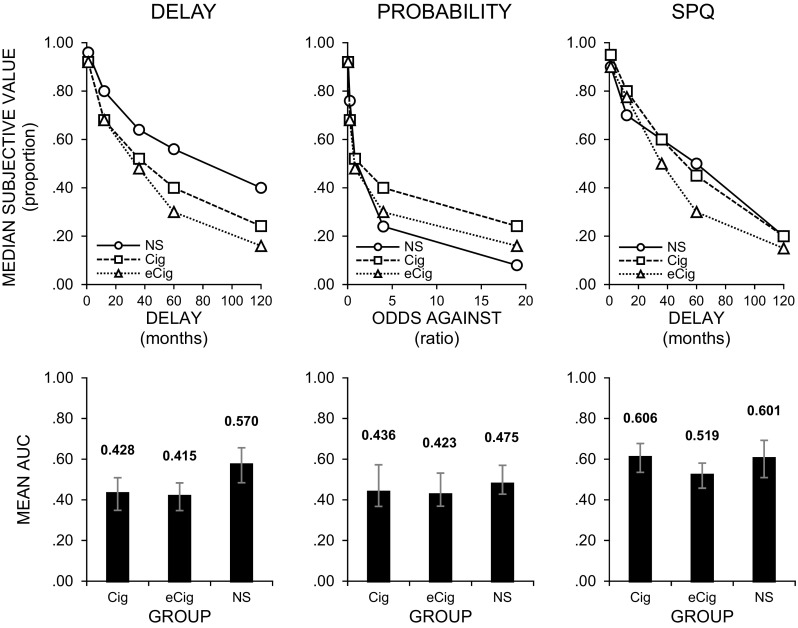
Median indifference points (*upper row*) with corresponding mean areas under the curve (AUC, *lower row*) for delay (*left column*) and probability (*center column*) discounting. Data points for SPQ (*right column*) refer to median estimations of subjective probability of obtaining delayed rewards (expressed in percentages and divided by 100) with the AUC computed as areas under the lines that connect these points (*lower row*). All data are presented for traditional cigarette smokers (Cig), e-cigarette users (eCig), and never smokers (NS). *Error bars* represent a 95% confidence interval for the mean

We observed significant differences between groups in rates of delay discounting [*F*(2, 87) = 5.090; *p* = 0.008; *η*_p_^*2*^ = 0.10] but not in the rate of probability discounting [*F*(2, 87) = 0.983; *p* = 0.378; *η*_p_^*2*^ = 0.02) or subjective probability of obtaining a delayed reward [*F*(2, 87) = 1.719; *p* = 0.185; *η*_p_^*2*^ = 0.04). In the delay condition, multiple comparisons with Sidak’s correction showed a significant difference between never smokers and traditional cigarette smokers (*p* = 0.032) and e-cigarette users (*p* = 0.015). However, there was no difference in rates of delay discounting between traditional cigarette smokers and e-cigarette users (*p* = 0.993). Mean values for the AUC in traditional cigarette smokers, e-cigarette users, and never smokers—with corresponding median indifference points—are presented in Fig. 1.

Table 2 illustrates the relationships between AUC measures for delay and probability discounting and SPQ scores in the overall sample and in subgroups. Overall, there was no significant correlation observed between delay and probability discounting. However, a positive correlation was found between delay discounting and SPQ score (i.e., the more participants discounted future delayed rewards, the less likely they perceived they would receive the reward). The same positive correlation between delay discounting and SPQ score was observed in the never smokers and traditional cigarette smoker groups. However, the correlation between delay discounting and SPQ score was absent in the in the e-smokers group. Finally, no correlation was observed between probability discounting and SPQ score in any of the subgroups nor across the sample overall.

**Table 2:** Pearson’s r correlation coefficients for delay discounting, probability discounting rate, and for SPQ (AUC measure) in the whole sample and across groups of never smokers, traditional cigarette smokers, and e-cigarette users

Measure	Delay	Probability
All participants		
Probability	0.177	-
SPQ	0.375**	0.048
Never smokers		
Probability	0.056	-
SPQ	0.495*	0.017
Cigarette smokers		
Probability	0.094	-
SPQ	0.446*	0.152
e-Cigarette users		
Probability	0.308	-
SPQ	0.017	0.114

*Note:* significant after applying Sidak’s correction for multiple comparisons: *p < 0.05; **p < 0.001

## Discussion

The primary aim of the present study was to compare delay discounting, probability discounting, and subjective probability of obtaining future rewards in three groups: cigarette smokers, e-cigarette users, and never smokers. The data suggest smokers (traditional cigarettes or e-cigarettes) discount delayed rewards more steeply than never smokers, but no difference in probability discounting was observed between groups. Furthermore, the present study did not find any differences in the estimation of subjective probability of obtaining a delayed reward among the three groups. Without a prior hypothesis or expectation, we found a positive correlation between delay discounting and SPQ scores that was present in the overall sample, traditional cigarette smokers, and never smokers. However, no correlation was observed in the e-cigarette users group which suggests SPQ scores may allow researchers to differentiate traditional cigarette smokers and e-cigarette users. However, until replicated, this observation needs to be used with caution.

There is large body of literature on delay and probability discounting and substance use and abuse (see Madden & Bickel, 2010). Among other things, studies show that nicotine addiction as manifested by smoking is predicted by delay, but not probability discounting (e.g., Mitchell, 1999; Ohmura, Takahashi, & Kitamura, 2005). Our results are consistent with previous studies that showed differences in the rate of delay discounting but not probability discounting when smokers and non-smokers are compared.

Contrary to Weidberg and colleagues (2016) and Chivers and colleagues (2016), we observed significantly steeper rates of delay discounting from participants in the e-smokers group compared to never-smoker control group. Weidberg and colleagues (2016) found that e-smokers discounted delayed rewards more steeply than former smokers and that smokers discounted more steeply than the control group, but observed no difference between e-smokers and the control group (with inclusion criteria of smoking less than 100 cigarettes before). Similarly, Chivers and colleagues (2016) found that e-cigarette users did not differ in rate of delay discounting from never smokers but that current cigarette smokers differed from never smokers. Finally, Weidberg and colleagues (2016) found that former smokers and control participants discounted rewards similarly which is consistent with previous research (e.g., Bickel, Odum, & Madden, 1999).

Informal comparison of discount rates between the three studies (present study; Chivers et al., 2016; Weidberg et al., 2016) suggests more similarity in results than null-hypothesis significance testing suggests. That is, visually, e-cigarette users’ rates of discounting are consistently steeper than controls and similar to cigarette smokers in all three studies. Relatedly, the rate of delay discounting for e-cigarette users in both previous studies was between traditional cigarette smokers and control groups, whereas no difference was observed between e-cigarette users and traditional cigarette smokers in the current study. In addition, participants in the Weidberg et al. (2016) study were included in the e-cigarette user group only if they had used e-cigarettes and not smoked traditional cigarettes in the previous 30 days. In contrast, participants in the current study self-reported that they were only e-cigarette users or only traditional cigarette smokers. As a result, some participants in the e-cigarette group could have smoked traditional cigarettes up to the day before the current study but were counted in the e-cigarette user group. This may have played a role in the similarity of discounting measures between traditional cigarette smokers and e-cigarette users as well as the significant difference in both of these groups compared to never smokers.

Closer inspection of Fig. 1 suggests there may have been a difference between never smokers and traditional cigarette smokers at high odds against (low probabilities). Specifically, never smokers were the most risk-averse and traditional cigarette smokers were the most risk-seeking. We analyzed this by using an exploratory non-parametric approach modified off the approach outlined by Yi, Chase, and Bickel (2007). All exploratory analyses resulted in no significant differences in probability discounting (nor in SPQ) between groups. However, the results of the AUC analysis relative to delay discounting were replicated.

The present study also provides some evidence that delay discounting and probability discounting are separate processes. We obtained significant differences in delay, but not probability discounting between groups. In addition, we observed no correlation between delay and probability discounting in contrast to a single trait view which predicts either a negative or positive correlation between delay and probability discounting (for example, see Białaszek et al., 2015; Mitchell, 1999, 2004; Ostaszewski, Green, & Myerson, 1998). Our study also supports the notion that delay and probability discounting might be separate but interacting processes in a non-uniform dimension (Cox & Dallery, 2016; Green, Myerson, & Vanderveldt, 2014; Vanderveldt, Green, & Myerson, 2015). We obtained differential levels of delay discounting across groups, and no unidirectional pattern of changes in all three dependent variables across groups. Largely, research that investigates both probability and delay discounting yields similar results (Mitchell, 1999; Ohmura, Takahashi, & Kitamura, 2005). For example, brief smoking abstinence led to steeper delay discounting but had no impact on probability discounting (Yi & Landes, 2012). Reynolds, Richards, Horn, and Karraker (2004) also showed significantly stronger differences between smokers and non-smokers in delay discounting, compared to probability discounting, which is partially in line with our results. In sum, impulsivity may be composed of separate but interacting processes. Future investigations using groups with different smoking status could compare discounting outcomes that are both delayed and risky—similar to those encountered in real-life situations.

No difference was found between smokers and never smokers in their perception of the certainty of future rewards as indicated by differences in AUC values. In a previous study by Reynolds, Patak, and Shroff (2007), adolescent smokers rated delayed rewards as less certain than non-smokers. The lack of consistency between our study and previous reports may be because perception of risk, and risk-taking itself, may change from adolescence to adulthood (Steinberg, 2004). The study by Reynolds, Patak, and Shroff (2007) was completed with adolescents, whereas the current study used adults.

We found an interesting pattern of correlations between delay discounting and SPQ scores. A positive correlation between the rate at which the participants devalued delayed rewards and their perceived certainty of future rewards was found in the overall sample, traditional cigarette smokers, and in never smokers—but not in e-cigarette users. This might suggest that the rate of reward devaluation by delay in e-smokers is independent of perceiving future events with varying degrees of certainty even though e-smokers value the probability of obtaining future rewards the same as non-smokers or traditional cigarette smokers. This pattern was found post-hoc, with no *a-priori* hypothesis, and may have been the result of the small sample size used in this study. Accordingly, it should be treated with caution until confirmed in further investigation. If confirmed, such results might suggest that measures of SPQ score can differentiate traditional cigarette smokers and e-cigarette users.

Several limitations to our results stem from how participants were assigned to groups. Participants were allocated to the different groups based only on verbal report of smoking as opposed to objective measures of smoking (e.g., CO levels in exhaled air, although this would be not indicative in e-smokers). Also, we did not control for using other nicotine products. It is widely recognized that many people who report using e-cigarettes use other nicotine products besides e-cigarettes (Saddleson et al., 2015). In addition, Kalkhoran and Glantz (2016) demonstrated that the odds of quitting when e-smoking are lower than the odds of quitting without using e-cigarettes.

As we mentioned, we observed a difference in FTND scores between two smoking groups. Goniewicz and colleagues (2014) provided further support that e-cigarettes do not serve as a cigarette substitute. They found a parallel increase in e-cigarette use and smoking prevalence from 2010 through 2013 when comparing data from two cross-sectional studies in Poland. Taken together, previous research suggests e-smokers may be more dependent on nicotine, which may be why we observed a significant difference in FTND scores between e-smokers and traditional cigarette smokers. Granted, an alternative explanation of the difference in FTND scores may be due to different patterns of nicotine consumption as one e-cigarette may not be equivalent to one cigarette smoked. Nevertheless, previous research suggests it is possible that nicotine dependence and nicotine levels were different between e-smokers and smokers in our study. This is a limitation because studies using non-human animals have shown that nicotine levels are related to measures of impulsivity (Anderson & Diller, 2010; Kelsey & Niraula, 2013). Recent analysis shows that the nicotine exposure remains unchanged in most people over time which potentially mitigates this limitation (Goniewicz et al., 2016). However, further studies should attempt to control for such variables as smoking history and the level of recent addiction.

Despite the declarative nature of the study, this study extends present knowledge by relating e-smoking to both delay discounting and probability discounting. This study also shows that measures such as the SPQ may provide additional insight to understanding addiction in those who use e-cigarettes.
